# Diabetic Pathophysiology Enhances Inflammation during Extracorporeal Membrane Oxygenation in a Rat Model

**DOI:** 10.3390/membranes11040283

**Published:** 2021-04-11

**Authors:** Yutaka Fujii, Takuya Abe, Kikuo Ikegami

**Affiliations:** 1 Department of Clinical Engineering and Medical Technology, Niigata University of Health and Welfare, Niigata 950-3198, Japan; takuya-abe@nuhw.ac.jp; 2 Department of Health and Medical Sciences, Chiba Institute of Science, Choshi 288-0025, Japan; kikegami@cis.ac.jp

**Keywords:** ECMO, diabetes, inflammatory response, rat ECMO model

## Abstract

Systemic inflammatory responses in patients undergoing extracorporeal membrane oxygenation (ECMO) contribute significantly to ECMO-associated morbidity and mortality. In recent years, the number of type 2 diabetes mellitus patients has increased, and the number of these patients undergoing ECMO has also increased. Type 2 diabetes mellitus is a high-risk factor for complications during ECMO. We studied the effects of ECMO on inflammatory response in a diabetic rat ECMO model. Twenty-eight rats were divided into 4 groups: normal SHAM group (normal rats: *n* = 7), diabetic SHAM group (diabetic rats: *n* = 7), normal ECMO group (normal rats: *n* = 7), and diabetic ECMO group (diabetic rats: *n* = 7). We measured the plasma levels of cytokines, tumor necrosis factor-α, and interleukin-6. Aspartate aminotransferase (AST), alanine aminotransferase (ALT), lactate dehydrogenase (LDH), blood urea nitrogen (BUN), creatinine (Cr), and liver-type fatty acid binding protein (L-FABP) were examined in the rat cardiopulmonary bypass model to ascertain organ damage. In addition, the lung wet-to-dry weight (W/D) ratio was measured as an index of pulmonary tissue edema. A pathologic evaluation of kidneys was conducted by hematoxylin-eosin (HE) and periodic-acid-methenamine-silver (PAM) staining. In the diabetic ECMO group, levels of cytokines, AST, ALT, LDH, and L-FABP increased significantly, reaching a maximum at the end of ECMO in comparison with other groups (*p* < 0.05). In addition, hematoxylin-eosin and periodic acid-methenamine-silver staining of renal tissues showed marked injury in the ECMO group (normal ECMO and diabetic ECMO groups). Furthermore, when the normal ECMO and diabetic ECMO groups were compared, severe organ injury was seen in the diabetic ECMO group. There was remarkable organ injury in the diabetic ECMO group. These data demonstrate that diabetes enhances proinflammatory cytokine release, renal damage, and pulmonary edema during ECMO in an animal model.

## 1. Introduction

Extracorporeal membrane oxygenation (ECMO) is known to cause a variety of complications while saving patients [1,2,3]. ECMO has multiple adverse effects with a systemic inflammatory response, mainly caused by blood coming in contact with the artificial foreign surface of the ECMO circuit [1,2,3]. This complex inflammatory chain reaction significantly affects the morbidity and mortality associated with extracorporeal life support (ECLS) [4].

In addition to contact with the ECMO unit, other potential factors involved in the inflammatory response include endotoxemia, ischemic reperfusion injury, and vascular hyperpermeability [2,3]. In addition, an increase in cytokines during ECMO aggravates the pro-inflammatory chain reaction [2,3,5]. Our recent study demonstrated that ECLS causes principal organ damage and a systemic inflammatory response in a small animal ECMO model [6,7,8]. ECMO has been commonly used in the world to support patients with severe pneumonia [9,10,11,12]. The active use of ECMO was also recommended for severe pneumonia from COVID-19, which has been rampant since 2020 [13].

Recently, the number of type 2 diabetes mellitus patients has been increasing, and the number of cardiac surgical procedures for these patients is also increasing [14]. Patients with diabetes experience increased activation of macrophages, which causes chronic inflammation [15,16]. ECLS in patients with diabetes may cause further inflammation. In the present study, ECMO’s effect was examined in a type 2 diabetic rat model.

In addition, the lung wet-to-dry weight (W/D) ratio was measured as an index of pulmonary tissue edema. A pathologic evaluation of kidneys by hematoxylin-eosin (HE) and periodic-acid-methenamine-silver (PAM) staining was also performed.

## 2. Materials and Methods

### 2.1. Animals

This study was performed in accordance with the National Institutes of Health’s guidelines for laboratory animal welfare. Sprague–Dawley rats (males, 14–16 w, 400–450 g, *n* = 14) and Spontaneously Diabetic Torii rats (males, 14–16 w, 400–450 g, *n* = 14) were housed 3 per cage under a 12 h light–dark cycle with food and water available ad libitum. All animals were provided by CLEA Japan, Inc. (Tokyo, Japan).

### 2.2. Anesthesia, Surgical Preparation, and ECMO

**Figure 1:**
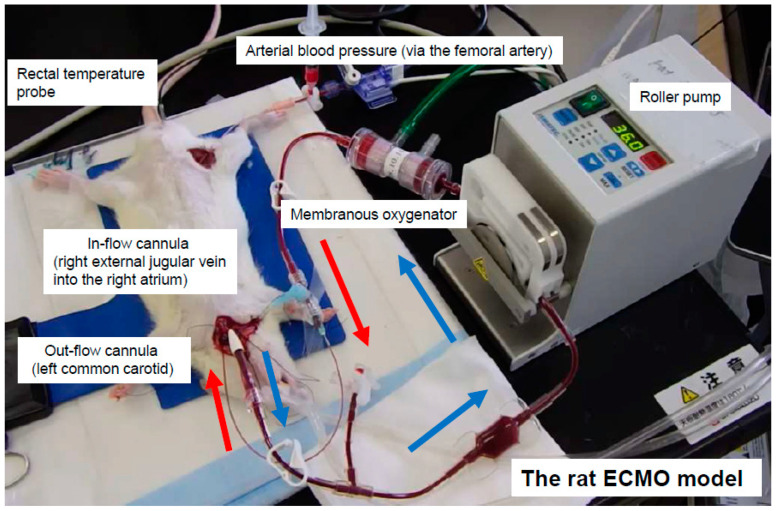
Extracorporeal circulation in a rat extracorporeal membrane oxygenation (ECMO) model.

After the rats were anesthetized by 4.5–5.0% isoflurane-mixed oxygen-enriched air inhalation with a vaporizer, they were placed in the supine position, and a rectal temperature probe was then inserted. The rats were orotracheally intubated using a 14 G cannula (Terumo Corp., Tokyo, Japan) and ventilated with a respirator for small animals (Model 683, Harvard Apparatus Ltd., Holliston, MA, USA). Ventilation was volume-controlled at a frequency of 70 breaths/min, a tidal volume of 10 mL/kg, and an inspired oxygen fraction of 40%. Anesthesia was maintained with 1.5–2.0% isoflurane (without neuromuscular blocking agents), and the rectal temperature was maintained at 35.5–36.5 °C throughout the experiment. The right femoral artery was cannulated with polyethylene tubing (inner diameter 0.5 mm, outer diameter 0.8 mm: Natsume Seisakusho Co. Ltd., Tokyo, Japan) for monitoring the arterial blood pressure using a Power-Lab (ML880, AD Instruments, Bella Vista, NSW, Australia). A polyethylene tubing (inner diameter 0.8 mm, outer diameter 1.2 mm: Natsume Seisakusho Co. Ltd., Tokyo, Japan) was used to cannulate the left common carotid artery as the outflow cannula for the ECMO system, as described in our previous reports [6,7,8]. Heparin sodium (500 IU/kg) was then administered through the outflow cannula. The right internal jugular vein was cannulated with A 16 G cannula with 4-side holes (depth 38 mm: Togomedkit Co., Ltd., Tokyo, Japan) for venous uptake. The same person conducted all experiments to standardize the procedure. The ECMO system was composed of a polyvinyl chloride tubing circuit (Senko Medical Co., Ltd., Tokyo, Japan), a specially designed membranous oxygenator for small animals (polypropylene, membrane area 0.03 m^2^: Senko Medical Co., Ltd., Osaka, Japan), and a mini roller pump (REGLO Digital ISM831, ISMATEC, Wertheim, Germany) primed by 7 mL of saline and 1 mL (1000 IU) of heparin. Figure 1 shows the experimental conditions.

### 2.3. Experimental Design

**Figure 2:**
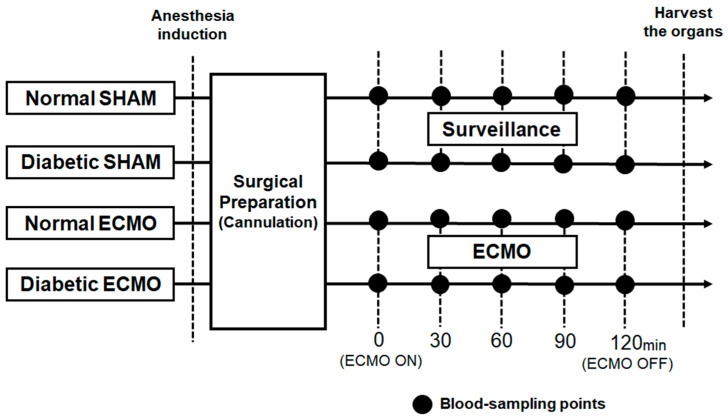
Schematic diagram of experimental design.

Twenty-eight rats were divided into 4 groups: normal SHAM group (normal rats: *n* = 7), diabetic SHAM group (diabetic rats: *n* = 7), normal ECMO group (normal rats: *n* = 7), and diabetic ECMO group (diabetic rats: *n* = 7). The normal SHAM and diabetic SHAM group only underwent surgical preparation without ECMO. The ECMO perfusion flow was initiated and maintained at 60–70 mL/kg/min. The arterial pressures of carbon dioxide (PaCO_2_) and oxygen (PaO_2_) were maintained at 35–45 mmHg and 250–350 mmHg, respectively, and managed with α-stat. Blood samples were collected at 5 defined time points, before ECMO (pre-ECMO) and 30 min, 60 min, 90 min, and 120 min after the initiation of ECMO (end-ECMO). Figure 2 shows the schematic diagram of the experimental design. Saline was used for fluid replacement management. Saline was injected in 1.0 mL increments at blood sampling (total injection volume: 5.0 mL during the experiment).

To evaluate the systemic inflammatory responses, tumor necrosis factor-α (TNF-α) and interleukin (IL)-6 were measured by an enzyme-linked immunosorbent assay (Bio-Plex Assay Kits, BIO-RAD Laboratories, Inc., Hercules, CA, USA). The concentrations of aspartate aminotransferase (AST), alanine aminotransferase (ALT), lactate dehydrogenase (LDH), blood urea nitrogen (BUN), and creatinine (Cr) were used to evaluate organ damage and were measured by automated colorimetry from blood plasma samples (DRI-CHEM 7000 Analyzer, FUJIFILM, Kanagawa, Japan). Liver-type fatty acid binding protein (L-FABP) was used as a biochemical marker for acute renal dysfunction [17] measured by a rat L-FABP ELISA kit (CMIC Co., Ltd., Tokyo, Japan).

Blood gases, pH, hemoglobin (Hb) concentration, and electrolytes were also measured (VetStat Electrolyte and Blood Gas Analyzer, IDEXX, Sydney, NSW, Australia). All animals were euthanized at the end of the experiment using a potassium chloride injection into the heart. The left lung was harvested and divided into 3 parts. The left lung superior-third was used for the calculation of the W/D ratio. The lung block was weighed before and after desiccation for 48 h in a dry oven at 70 °C [6,7].

In addition, 3-μm thick, formalin-fixed, paraffin-embedded sections were stained with HE and PAM to assess renal pathology at the end of the experiment.

### 2.4. Statistics

All data are expressed as means ± standard error (SE). The comparisons among groups were performed by analysis of variance. The Fisher protected least significant difference (PLSD) post hoc test was used for subsequent comparisons between groups at the same time points. All statistical analyses were performed using Stat View 5.0 (Abacus Concepts, Berkeley, CA, USA). Additionally, *p* < 0.05 was considered statistically significant.

## 3. Results

Table 1 shows the changes in hemodynamic variables, Hb concentration, PaO_2_, and PaCO_2_ in each group. Mean arterial pressure (MAP) and Hb were significantly decreased during ECMO in both normal and diabetic ECMO groups.

The PaO_2_ levels were significantly higher (*p* < 0.05) in the ECMO group (normal ECMO and diabetic ECMO groups) than in the SHAM group (normal SHAM and diabetic SHAM groups), although the differences were not statistically significant. In contrast, no statistical difference was found in the PaCO_2_ level between these groups.

Before ECMO, plasma levels of cytokines were not significantly different among the normal SHAM group, diabetic SHAM group, normal ECMO group, or diabetic ECMO group. However, Cr, BUN, and L-FABP were significantly higher in the diabetic groups (diabetic SHAM and diabetic ECMO groups) than in the normal groups (diabetic SHAM and diabetic ECMO groups) before ECMO. Plasma levels of cytokines, AST, ALT, LDH, Cr, BUN, and L-FABP remained unchanged during the experimental period in the SHAM groups (normal SHAM and diabetic SHAM groups). In the diabetic ECMO group, pro-inflammatory cytokines increased significantly, reaching a maximum at the end of ECMO (TNF-α, 1576 ± 255 pg/mL; IL-6, 2620 ± 599 pg/mL).

**Figure 3:**
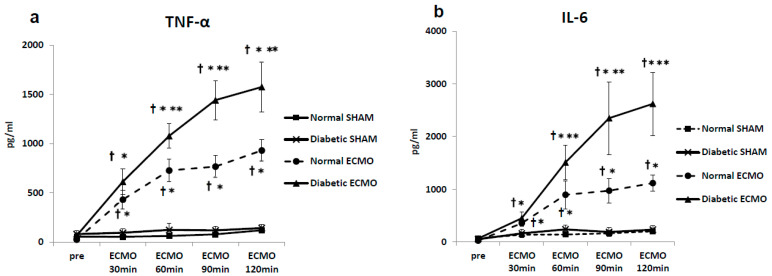

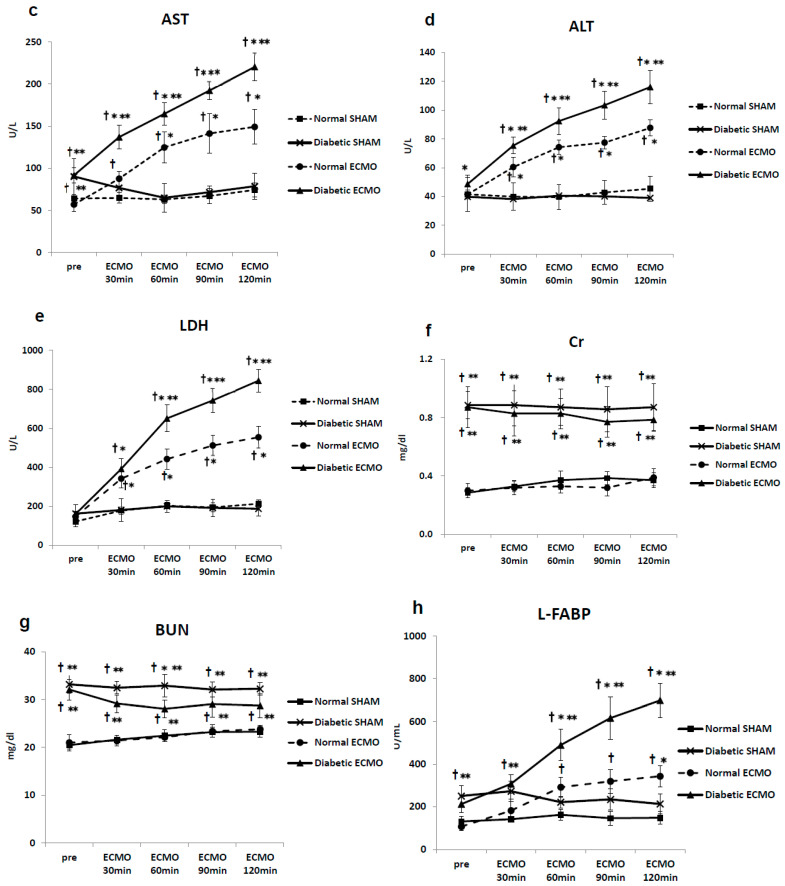
Plasma tumor necrosis factor (TNF)-α (**a**), interleukin (IL)-6 (**b**), aspartate aminotransferase (AST) (**c**), aminotransferase (ALT) (**d**), lactate dehydrogenase (LDH) (**e**), creatinine (Cr) (**f**), blood urea nitrogen (BUN) (**g**), and liver-type fatty acid binding protein (L-FABP) (**h**). ^†^ *p* < 0.05 versus normal SHAM group at the same time point, * *p* < 0.05 versus diabetic SHAM group at the same time point, ** *p* < 0.05 versus normal cardiopulmonary bypass group at the same time point.

In the diabetic ECMO group, levels of AST, ALT, LDH, and L-FABP increased significantly, reaching a maximum at the end of ECMO in comparison with other groups (LDH, 845 ± 158 U/L; AST, 220 ± 17 U/L; ALT, 116 ± 12 U/L; L-FABP, 698 ± 80 U/mL). In terms of Cr and BUN, the diabetic groups (diabetic SHAM group and diabetic ECMO group) showed significantly higher levels (*p* < 0.05) during the experimental period (Figure 3).

**Figure 4:**
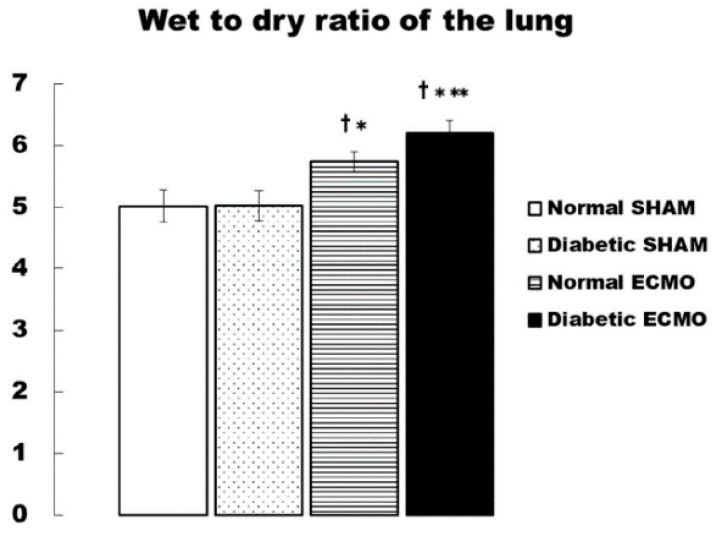
Wet-to-dry (W/D) ratio of the left lung at the end of cardiopulmonary bypass. ^†^ *p* < 0.05 versus normal SHAM group at the same time, * *p* < 0.05 versus diabetic SHAM group at the same time, ** *p* < 0.05 versus normal ECMO group at the same.

The W/D ratio was much higher in the ECMO groups (normal ECMO and diabetic ECMO groups) than in the SHAM groups (normal SHAM and diabetic SHAM groups). Additionally, the diabetic ECMO group showed a significantly higher W/D ratio than the normal ECMO group (normal ECMO group, 5.74 ± 0.16 vs. diabetic ECMO group, 6.20 ± 0.20 (*p* < 0.05) (Figure 4).

**Figure 5:**
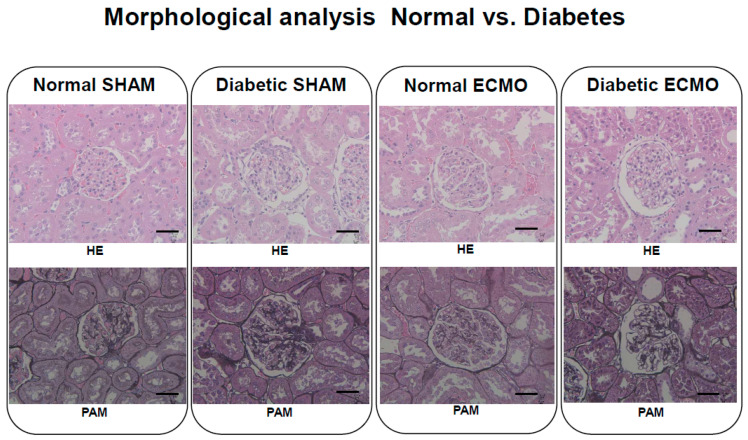
Pathological image of renal tissue (at 200-fold magnification). HE, hematoxylin-eosin; PAM, periodic acid-methenamine-silver stain. Scale bar: 50 μm. Normal SHAM: Intact. Diabetic SHAM: Slight cytolysis was shown, and the organ structure was maintained. Normal ECMO: Cell injury was similar to the level of diabetic SHAM rats. Diabetic ECMO: Tubular epithelium was desquamated from the basement membrane.

The extent of renal tubular damage assessed by histologic analyses is shown in Figure 5. The HE and PAM staining of renal tissues showed marked injury in the ECMO group (normal ECMO and diabetic ECMO groups). Furthermore, when the normal ECMO and diabetic ECMO groups were compared, the injury was severe in the diabetic ECMO group. Specifically, glomerular atrophy and severe tubular injury were observed in the diabetic ECMO group.

## 4. Discussion

It is well known that ECLS causes a pro-inflammatory chain reaction via blood interactions with the artificial extracorporeal device [18,19]. The present study using an experimental rat model also demonstrated that ECMO treatment increased plasma levels of the inflammatory markers, TNF-αand IL-6, in the normal SHAM group, as shown in Figure 3. The systemic inflammatory response may be critically important for the perioperative outcome [20,21]. Furthermore, in recent years, cardiovascular surgery for patients with diabetes has been increasing [22]. The diabetic population accounts for more than 38% of patients undergoing cardiac operations, especially coronary revascularization, in the world [22].

In the present study, we examined the effect of a type 2 diabetic setting during ECMO treatment on the inflammatory response in an animal model. The major finding of this study was that rats with diabetes in the ECMO model showed rapidly increased inflammatory cytokines, such as TNF-α and IL-6, and other measurements, such as AST, ALT, LDH and L-FABP. There were no differences in inflammatory cytokines and all biochemical data between the diabetic SHAM and diabetic ECMO groups before the experiment. The rapidly increased inflammatory cytokines and all biochemical data imply that these findings are due to ECMO. The pulmonary tissues of the rats in the diabetic ECMO group had higher W/D ratios at the end of ECMO than the other groups, and, therefore, they were presumed to have accumulated more water. Furthermore, in the diabetic ECMO group, it is presumed that renal damage was caused by a sharp rise in the acute renal failure markers, L-FABP, severe tubular damage, and glomerular atrophy.

People with diabetes and other lifestyle-related diseases are prone to arteriosclerosis, and mast cells in these patients are thought to cause cell death and chronic inflammation [23]. ECMO further enhances the inflammatory response and likely induces a rapid release of cytokines. Furthermore, it is well known that vascular endothelial dysfunction resulting in ischemia occurs in vulnerable organs in the diabetic setting [24,25]. In particular, the kidney is susceptible to damage by ECLS [26,27]. In this study, non-physiologic circulation by ECMO was considered closely associated with acute kidney injury (AKI). It has also been reported that mesenteric endothelial dysfunction occurs in animal experiments using a diabetes model [28]. Furthermore, a previous study showed the effects of green tea polyphenols on acute kidney injury after cardiopulmonary bypass in diabetes rats [29].

In addition, it was observed that the tendency for pulmonary edema was severe in the diabetes groups, and vascular permeability was enhanced. Previously, the relationship between diabetic pathogenesis and oxidative stress has attracted attention [30,31]. There are some reports that patients with diabetes are susceptible to oxidative stress leading to inflammation and organ damage during cardiac surgery [32,33]. Typically, however, arterial pressure of oxygen is maintained at high levels in clinical ECLS [34]. Our previous study also showed that oxidative stress generates superoxide was redundant to the systemic inflammatory response in ECLS [7]. Several studies have shown that the ECMO circuit’s inner side of the walls activates leukocytes, platelets, and the complement system. Activated leukocytes release cytotoxic agents and reactive oxygen species associated with the systemic inflammatory response and organ damage [35,36]. Our previous study showed that selective reduction of hydroxyl radicals with hydrogen gas attenuates both pro-inflammatory cytokines—TNF-α and IL-6—suggesting that this radical acts to non-selectively increase these cytokines [6]. The present animal study demonstrated that a diabetic setting causes the elevation of cytokines and organ damage in the lungs and kidneys in comparison with the normal group treated with ECMO. Future studies focusing on oxidative stress in diabetic patients during ECMO should enable us to more deeply understand the sequential mechanism. In addition, aggressive leukocyte and cytokine removal therapies should be considered to suppress the inflammation during ECMO. Various leukocyte depletion filters have been developed in the field of cardiopulmonary bypass [37]. Furthermore, polymethyl methacrylate materials are known to remove cytokines [38]. Applying this technology to ECMO will lead to inflammation suppression.

Recently, new biomarkers for the early detection of AKI have been attracting attention [17,39]. In our present study, L-FABP was observed to increase in the diabetic groups immediately after ECMO induction. The research model used in the present study may help clarify the mechanism of injury in diabetic patients undergoing ECMO and suggest treatment and preventive measures for this injury in the future.

## 5. Conclusions

In conclusion, we report that ECMO treatment enhances the systemic release of proinflammatory cytokines and causes renal damage and pulmonary edema in rats with diabetes. These conditions are associated with a systemic inflammatory process and may result from un-physiologic tissue perfusion. Principal organ injury may play a key role in the development of multiple organ failure after ECMO and trigger higher postoperative mortality and perioperative complications that occur in diabetic patients undergoing ECMO.

## Figures and Tables

**Table 1:** Hemodynamic variables, blood gas partial pressures, hemoglobin, and pH before and during ECMO.

Variable	Group	Pre-ECMO	ECMO 60 min	ECMO 120 min
MAP (mmHg)	Normal SHAM	99 ± 3	92 ± 6	84 ± 5
	Diabetic SHAM	94 ± 3	88 ± 4	83 ± 5
	Normal ECMO	97 ± 3	76 ± 5 ^†^*	75 ± 4 ^†^*
	Diabetic ECMO	96 ± 2	79 ± 4 ^†^*	77 ± 4 ^†^*
HR (beat/min)	Normal SHAM	368 ± 12	366 ± 11	374 ± 5
	Diabetic SHAM	353 ± 10	349 ± 11	360 ± 9
	Normal ECMO	375 ± 11	369 ± 14	343 ± 7 ^†^*
	Diabetic ECMO	362 ± 11	357 ± 18	346 ± 14 ^†^*
PaO_2_ (mmHg)	Normal SHAM	98 ± 2	98 ± 2	102 ± 4
	Diabetic SHAM	100 ± 2	99 ± 2	98 ± 1
	Normal ECMO	100 ± 4	301 ± 21 ^†^*	290 ± 19 ^†^*
	Diabetic ECMO	102 ± 4	289 ± 20 ^†^*	295 ± 17 ^†^*
PaCO_2_ (mmHg)	Normal SHAM	40 ± 2	40 ± 1	38 ± 3
	Diabetic SHAM	39 ± 1	38 ± 2	39 ± 1
	Normal ECMO	40 ± 1	39 ± 1	38 ± 1
	Diabetic ECMO	41 ± 1	39 ± 1	38 ± 1
Hb (g/dL)	Normal SHAM	14.1 ± 0.5	13.9 ± 0.5	13.0 ± 0.6
	Diabetic SHAM	14.0 ± 0.4	12.6 ± 0.5	12.9 ± 0.5
	Normal ECMO	14.4 ± 0.2	10.4 ± 0.5 ^†^*	10.2 ± 0.5 ^†^*
	Diabetic ECMO	14.0 ± 0.2	10.3 ± 0.7 ^†^*	10.5 ± 0.5 ^†^*
pH	Normal SHAM	7.35 ± 0.03	7.37 ± 0.03	7.40 ± 0.03
	Diabetic SHAM	7.36 ± 0.02	7.37 ± 0.03	7.39 ± 0.02
	Normal ECMO	7.37 ± 0.02	7.38 ± 0.02	7.39 ± 0.03
	Diabetic ECMO	7.37 ± 0.02	7.40 ± 0.01	7.40 ± 0.02

Variables are expressed by mean ± standard error. ^†^ *p* < 0.05 versus Normal SHAM group at the same time, * *p* < 0.05 versus Diabetic SHAM group at the same time. MAP: mean arterial pressure, HR: heart rate, PaO_2_: partial pressure of arterial oxygen, PaCO_2_: partial pressure of arterial carbon dioxide_,_ Hb: hemoglobin, and pH: power of hydrogen.

## Limitations

There are several limitations to the present study. First, we evaluated parameters during short-term ECMO. Further studies are needed to assess the systemic inflammatory response and organ damage during long-term ECMO and after weaning ECMO. Second, this study was not able to observe differences due to the severity of diabetes. Symptoms of diabetes are variable and vary from patient to patient. Future studies will allow us to elucidate the sequential mechanisms of the pathologic condition by evaluating the histopathological severity of diabetes.

## Abbreviations

ECMO	Extracorporeal membrane oxygenation
ECLS	Extracorporeal life support
PaO_2_	Arterial pressure of oxygen
PaCO_2_	Arterial pressure of carbon dioxide
TNF-α	Tumor necrosis factor-α
IL	Interleukin
AST	Aspartate aminotransferase
ALT	Alanine aminotransferase
LDH	Lactate dehydrogenase
BUN	Blood urea nitrogen
Cr	Creatinine
L-FABP	Liver-type fatty acid binding protein
Hb	Hemoglobin
HE	Hematoxylin-eosin
PAM	Periodic-acid-methenamine-silver stain
W/D	Wet-to-dry
SE	Standard error
ANOVA	Analysis of variance
PLSD	Protected least significant difference
